# Eating before Sleeping

**Published:** 1890-02

**Authors:** 


### Eating Before Sleeping.

A recent writer says that the view that brain workers should go supperless to bed is not good advice. Most medical authorities of the day think it wrong. It is a fruitful source of insomnia and neurasthenia. The brain becomes exhausted by its evening work and demands rest and refreshment of its wasted tissues, not by indigestible salads and "fried abominations," but by some nutritious, easily digested and assimilated articles. A bowl of stale bread and milk, of rice, or some other farinaceous food, with milk or hot soup, would be more to the purpose. Any of these would insure a sound night's sleep, from which a man would awaken refreshed.

